# Head-and-neck squamous cell carcinoma risk in smokers: no association detected between phenotype and *AHR*, *CYP1A1*, *CYP1A2*, or *CYP1B1* genotype

**DOI:** 10.1186/s40246-016-0094-y

**Published:** 2016-11-28

**Authors:** Lucia F. Jorge-Nebert, Ge Zhang, Keith M. Wilson, Zhengwen Jiang, Randall Butler, Jack L. Gluckman, Susan M. Pinney, Daniel W. Nebert

**Affiliations:** 1 Department of Environmental Health and Center for Environmental Genetics, University of Cincinnati Medical Center, Cincinnati, OH 45267-0056 USA; 2 Division of Human Genetics, Department of Pediatrics & Molecular Developmental Biology, Cincinnati Children’s Hospital, Cincinnati, Ohio 45229-2899 USA; 3 Department of Otolaryngology-Head and Neck Surgery, University of Cincinnati College of Medicine, Cincinnati, OH 45267-0528 USA; 4 Department of Pathology and Laboratory Medicine, University of Cincinnati College of Medicine, Cincinnati, OH 45267-0533 USA; 5 Present address: Genesky Diagnostics, Suzhou, China

**Keywords:** *AHR* gene, *CYP1A1*, *CYP1A2*, *CYP1B1* genes, Tag-SNPs (single nucleotide polymorphisms), Head-and-neck squamous cell carcinoma (HNSCC), Cigarette smoking, Extreme discordant phenotype method, Population stratification, Candidate-gene approach to genotype-phenotype association

## Abstract

**Background:**

Head-and-neck squamous cell carcinoma (HNSCC) differs between smokers and nonsmokers in etiology and clinical presentation. Because of demonstrated unequivocal involvement in smoking-induced cancer in laboratory animals, four candidate genes––*AHR*, *CYP1A1*, *CYP1A2*, and *CYP1B1*––were selected for a clinical genotype-phenotype association study of HNSCC risk in smokers. Thirty-six single-nucleotide variants (mostly tag-SNPs) within and near these four genes [16 (*AHR*), 4 (*CYP1A1*), 4 (*CYP1A2*), and 12 (*CYP1B1*)] were chosen.

**Methods:**

Extreme discordant phenotype (EDP) method of analysis was used to increase statistical power. HNSCC patients––having smoked 1–40 cigarette pack-years––represented the “highly-sensitive” (HS) population; heavy smokers having smoked ≥80 cigarette-pack-years without any type of cancer comprised the “highly-resistant” (HR) group. The vast majority of smokers were intermediate and discarded from consideration. Statistical tests were performed on *N* = 112 HS and *N* = 99 HR DNA samples from whole blood.

**Conclusions:**

Among the four genes and flanking regions––one haploblock, ACTTGATC in the 5′ portion of *CYP1B1*, retained statistical significance after 100,000 permutations (*P* = 0.0042); among our study population, this haploblock was found in 36.4% of African-American, but only 1.49% of Caucasian, HNSCC chromosomes. Interestingly, in the 1000 Genomes Project database, frequency of this haplotype (in 1322 African and 1006 Caucasian chromosomes) is 0.356 and 0.003, respectively. This study represents an excellent example of “spurious association by population stratification”. Considering the cohort size, we therefore conclude that the variant alleles chosen for these four genes, alone or in combinations, are not statistically significantly associated with risk of cigarette-smoking-induced HNSCC.

**Electronic supplementary material:**

The online version of this article (doi:10.1186/s40246-016-0094-y) contains supplementary material, which is available to authorized users.

## Introduction

Worldwide, head-and-neck squamous cell carcinoma (HNSCC) is the sixth most common cancer. An increased risk of HNSCC among cigarette smokers is well known. In addition, high-risk types of human papilloma virus (HPV) are associated with certain HNSCCs, specifically a subset arising in the oropharynx. It also appears very likely that there exists a genetic predisposition to smoking-induced HNSCC risk. Clearly, “cancer” represents a multifactorial trait involving hundreds, if not thousands, of genes, plus epigenetic and environmental effects. It remains possible, however, that—if a candidate-gene approach that embraces a method having sufficient statistical power is applied to a sufficiently large cohort—then a genotype-phenotype association might be demonstrated for one or more “small-effect” genes. This study describes an attempt to establish such an association.

Hundreds of polycyclic aromatic hydrocarbons (PAHs) are present in cigarette smoke. Many laboratory animal studies have demonstrated that aryl hydrocarbon receptor (AHR)-regulated cytochrome P450 family-1 (CYP1) enzymes (CYP1A1, CYP1A2, and CYP1B1) metabolize PAHs to reactive oxygenated intermediates. When cancer initiation occurs via “direct contact” with a carcinogen, e.g., cigarette smoke, we believe that HNSCC will more likely be associated with CYP1-mediated metabolic activation [5, 27, 38], compared with a distal cancer site such as kidney [27].

### Description of the four candidate genes

*AHR* codes for a ligand-activated transcription factor controlling numerous genes and critical cell pathways [40], including up-regulation of *CYP1A1*, *CYP1A2*, and *CYP1B1* genes [33]. AHR foreign ligands include chemicals such as PAHs; polyhalogenated dibenzo-*p*-dioxins, dibenzofurans and biphenyls; and benzoflavones found especially in cruciferous plants [26]. AHR endogenous ligands include indoles and tryptophan-derived moieties and an unknown number of the >150 members of the lipid mediator second-messenger family [7, 32]. The highly conserved AHR exists in all vertebrates and has also been reported to exist—without ligand-binding properties—in mollusk, *Caenorhabditis elegans*, and *Drosophila* [1].

*CYP1A1* encodes the P450 monoxygenase that metabolizes planar substrates, many of which are PAHs and biphenyls. CYP1A1 metabolizes few, if any, drugs. Decades of PAH-treated lab animal studies have shown strong correlations of inducible CYP1A1 with various types of cancer—in tissues in contact with the administered PAH [27]. Although basal CYP1A1 expression in animal and human tissues is nearly always nil, inducible CYP1A1 activity is ubiquitous, located in virtually every tissue and cell type of the body. For example, inducible CYP1A1 is found in white blood cells, endothelial cells of blood vessels, lung, kidney, skin, and epithelial lining of the head and neck and upper and lower gastrointestinal (GI) tract. Inducible CYP1A1 also is seen early in embryogenesis [30].

*CYP1A2* codes for the CYP1A2 monooxygenase that metabolizes about two dozen drugs—including caffeine and theophylline—plus many environmental aromatic amines. Substantial basal (constitutive) CYP1A2 activity occurs in mammalian liver. Whereas >60-fold differences in human hepatic CYP1A2 (mRNA, protein, and activity) exist between individuals in any population studied, etiology remains unknown. Human *CYP1A2* gene expression is not detectable in embryo, fetus,﻿ or kidney but is inducible by PAHs mostly in the liver, GI tract, pancreas, nasal epithelium, brain, and lung [30].

*CYP1B1* encodes the CYP1B1 monooxygenase which, like CYP1A1, metabolizes numerous PAHs and biphenyls, *N*-heterocyclic amines, arylamines and amino azo dyes, and other carcinogenic and toxic environmental chemicals. Also, like CYP1A1, CYP1B1 metabolizes few, if any, drugs. Unlike CYP1A1, CYP1B1 often exhibits substantial basal levels (e.g., endocrine tissues, tumors). CYP1B1 expression is induced in vascular endothelial cells, thymus/marrow and immune cells, breast, prostate, uterus, epithelial lining of the head and neck and upper GI tract, and various types of cancers [30].

Mouse *Ahr* knockouts and all three *Cyp1* single-, plus all three double- and the triple-knockout lines are viable and able to reproduce––although serious problems occur in *Ahr(−/−)* knockout [10, 20] and in *Cyp1a1/1a2/1b1(−/−)* triple-knockout mice [8, 31]. Whereas no human “knockout” equivalent has been found for *AHR*, *CYP1A1*, or *CYP1A2*, null mutations in *CYP1B1* are associated with primary congenital glaucoma [44], suggesting that, during embryogenesis, development of the eye’s anterior chamber requires metabolism of a critical endogenous CYP1B1 substrate, most likely a lipid mediator [34].

### EDP method

Given any gradient for a multifactorial trait, if one selects the two extremes of the phenotypic gradient [28] and disregards intermediate responders in whom genes contributing to phenotype are likely to overlap—then statistical power can be increased [46]. Among HNSCC patients, we selected those with a history of 1–40 cigarette pack-years (Cig-Pk-Yrs) as “highly sensitive” (HS). In the same clinic, we selected heavy smoker volunteers with ≥80 Cig-Pk-Yrs having no types of cancer, as “highly resistant” (HR) controls. All nonsmokers, smokers intermediate between HS and HR criteria, and other patients were excluded from our study.

### SNP typing of the four genes

The human *AHR* gene is located at chromosome (Chr) 7p15 and *CYP1B1* gene at Chr 2p22.2. The *CYP1A2_CYP1A1* locus, on Chr 15q24.1, contains the two genes oriented head-to-head with a bidirectional promoter [6]. The purpose of this study was to search for single single-nucleotide polymorphism (SNP) marker and haplotype associations in these four selected genes that might be statistically significantly correlated with greater risk of HNSCC in HS cancer patients, compared with HR heavy smokers having no cancer.

## Methods

### Clinical screening and patient/volunteer recruitment

HS patients were identified at the Barrett Cancer Center (Department of Otolaryngology-Head and Neck Surgery, University of Cincinnati College of Medicine) and the Cincinnati Veterans’ Association (VA) Hospital. Additional patients were identified within the Fernald Community Project. HR volunteers were recruited at the Barrett Cancer Center, Cincinnati VA Hospital, Fernald Project, and throughout the community in response to flyers. A questionnaire was used to identify HNSCC patients—who qualified as HS because they had smoked 1–40 Cig-Pk-Yrs (e.g., “20 Cig-Pk-Yr” denotes someone who might have smoked one pack per day for 20 years or one-half pack per day for 40 years). Volunteers, having no cancer of any kind (with exception of UV-caused skin cancers), despite having smoked ≥80 Cig-Pk-Yrs, qualified for the HR group and were also identified by questionnaire.

The Fernald Community blood samples originated from an earlier independent study, as described [39]. At all times, we followed the clinical protocol—titled “Human Cancer and *AHR/CYP1A1/1A2/1B1* Gene Polymorphisms” (#03-08-07-01)—annually approved for the entire study, without any HIPAA issues or concerns, by the University of Cincinnati Medical Center Institutional Review Board (IRB).

### Questionnaires

Questions asked of all participants included self-identified ethnicity, pipe or cigar smoking, second-hand smoke, high-tar vs. low-tar cigarettes, alcohol consumption, use of snuff/chewing tobacco, amount of coffee consumed, occupational hazards, consumption and amount of daily antioxidants, frequency of eating grilled meat, and family history. Staff administering the questionnaire ranked these eleven caveats as zero (little or negligible contact), one (intermediate contact or exposure), or two (high exposure). A copy of this questionnaire is included as Additional file 1.

### Clinical samples

Whole blood for DNA isolation was collected from HS and HR subjects via antecubital venipuncture. BD Vacutainer® systems (Becton, Dickinson and Co.; Franklin Lakes, NJ) were used. We collected two 13 × 100 mm Vacutainer® K_2_EDTA tubes (lavender caps) for a total volume of 10 mL. Freshly collected whole blood was refrigerated at 4 °C and kept not longer than 24 h at that temperature. If genomic DNA (gDNA) was not isolated from blood within 24 h of collection, the blood was stored at −80 °C usually for not longer than 8 weeks and then thawed the same day the isolation procedure was performed.

### DNA preparation

For the 10 mL of blood—collected at the Barrett Cancer Center and Cincinnati VA Hospital—gDNA was isolated using the QIAamp® DNA Blood Maxi kit (QIAGEN, Hilden, Germany) by applying the spin protocol. Whole-blood samples from the Fernald Community cohort, which had been stored at −70 °C, were thawed on the day of isolation. Average volume of these samples was 2 mL; gDNA was therefore isolated using the QIAamp® DNA Blood Midi kit (QIAGEN) and spin protocol. Both QIAGEN kits employed special patented columns for purification of gDNA present in blood pretreated with proteases, in a highly denaturing medium containing guanidine hydrochloride.

Highly purified gDNA isolated in this fashion was quantified by UV absorption at 260 nm using a mini-spectrometer (NanoDrop® ND-1000, Thermo Scientific; Waltham, MA). Purity was evaluated using the 260/280 and 260/230 absorbance ratios.

### Selection of common tag-SNPs

Tag-SNPs and other variants of interest were chosen in order to study variation within each of the genetic loci and to correlate these variations within each person in the HS and HR cohorts. Tag-SNPs were selected by the HaploView program [3], using a MAF cutoff of 10.0% and an *r* ^2^ threshold of 0.8. We used previous sequencing data of the *CYP1* locus (~40 kb) which includes the 23.3-kb bidirectional promoter [14, 15]. For *AHR* and *CYP1B1*, we selected tag-SNPs from targeted sequencing, including 10 kb of 5′ flanking regions in 24 Caucasian DNA samples (Z.J., *data not shown*). The ultimate total was 36 tag-SNPs: 16 for *AHR*, 4 for *CYP1A1*, 4 for *CYP1A2*, and 12 for *CYP1B1*.

These tag-SNPs are in linkage disequilibrium (LD) with other variants in their vicinity. Thus, their selection and genotyping were expected to simplify studies of the four candidate genes—by reducing the number of variants needed for a comprehensive study. Whole-genome sequencing was not yet an option at the time of this study.

### Genotyping of tag-SNPs

Tag-SNP genotypes for each HS and HR individual were determined using the ABI Prism® SnaPshot^TM^ Multiplex system, which allows for the typing of up to ten SNPs simultaneously. Chemistry of the applied kit is based on the dideoxy single-base extension of unlabeled primer or primers, catalyzed by AmpliTaq DNA polymerase, FS; this permits incorporation of a single fluorescently-labeled ddNTP on the 3′-end of the primer.

### Statistical analyses of genotype-phenotype association

Bioinformatics software HaploView (v. 4.2, Mark Daly’s lab; MIT/Harvard Broad Institute) was used to determine associations between the markers—variation in SNPs—and phenotype in HS vs. HR samples. This software also analyzes LD patterns, generating haploblocks, and inferring haplotypes. Criteria applied to determine haploblocks within genes was the “solid spine of LD”. Associations between these haplotypes and phenotypes were also calculated via the chi-square association test of every haplotype (exhaustive haplotype test). In addition, we applied the permutations test (results randomized 100,000 times) to adjust for multiple tests of multiple SNPs or haplotypes. Haplotype estimates for each individual HS and HR were obtained through the software program phase (v. 2.1.1, Matthew Stephens’s lab; University of Chicago). Differences in frequencies of haplotypes between populations were examined for significance using Fisher’s exact test.

## Results

HNSCC in the present study refers exclusively to squamous cell carcinoma of the oral cavity, larynx, oropharynx, and hypopharynx. Tumors of the face, salivary glands, nasopharynx, and brain were excluded. A substantial number (~15%) of HNSCC patients have no cigarette smoking history. Clinical and genetic features of HNSCC in nonsmokers, former smokers, and current smokers are known to be distinctly different [17]. Compared with malignancies in nonsmokers, smokers exhibit more tumors of the larynx, hypopharynx, and floor of the mouth; a much greater *TP53* mutation rate; a substantially higher percent of loss of heterozygosity at Chr 3p, 4q, and 11q13; and a greater overall average number of chromosomal losses. In contrast, the percentage with HPV infection was marginally, but not statistically, lower in nonsmoker malignancies [17].

We excluded all nonsmokers (i.e., those having smoked no more than one Cig-Pk-Yr, i.e., <7300 cigarettes, <365 packs, over a lifetime) because the mechanism of HNSCC tumorigenesis is likely not to be relevant to activation by cigarette smoke PAHs involving any of the four genes under study. It should be noted that more than 85% of all HNSCC smokers did not qualify because they had smoked >40 Cig-Pk-Yrs and <80 Cig-Pk-Yrs; this selective stringency creates a more robust statistical power [46], but unfortunately fewer numbers of qualified individuals.

Over a 5-year period, we collected gDNA from a total of 149 blood samples from the Barrett Cancer Center and Cincinnati VA Hospital, plus 62 samples from the Fernald Project. Of the 211 total samples, there were 94 and 18 HS patients from the Barrett Center/VA Hospital and Fernald Project, respectively, and 55 and 44 HR samples from the Barrett Center/VA Hospital and Fernald Project, respectively. Total subjects having high-quality gDNA ultimately included 112 HS and 99 HR for this study (Table 1).

**Table 1:** Demographics of entire cohort studied

Ethnicity (self-identified)					
	HS	HR	Total	Percent	
Caucasian-American	101	95	196	92.9	
African-American	11	3	14	6.6	
Latino-American	0	1	1	0.5	
Total	112	99	211	100	
By site of collection					
Ethnicity (self-identified)	Barrett/VA		Fernald		Total
	HS	HR	HS	HR	
Caucasian-American	83	51	18	44	196
African-American	11	3	0	0	14
Latino-American	0	1	0	0	1
Total	94	55	18	44	211

### Statistical analyses of covariates

With regard to the 11 covariates included in the questionnaire (Appendix 1; *described above*), we specifically focused on occupational history, amount of dietary grilled meat, and family history of cancer (ranked as “0,” “1,” or “2” for each individual); the other 8 parameters did not have sufficient information for a substantial number of participants. We used logistic regression and random-forests models for statistical analysis of possible associations between the selected best SNP markers and these three covariates. No statistically significant associations or trends of association (*P* < 0.05) were found between any of these covariates and phenotype (*data not shown*). The “self-identified ethnicity” was important and is discussed in great detail below.

### Genotype-phenotype association analysis

**Fig. 1:**
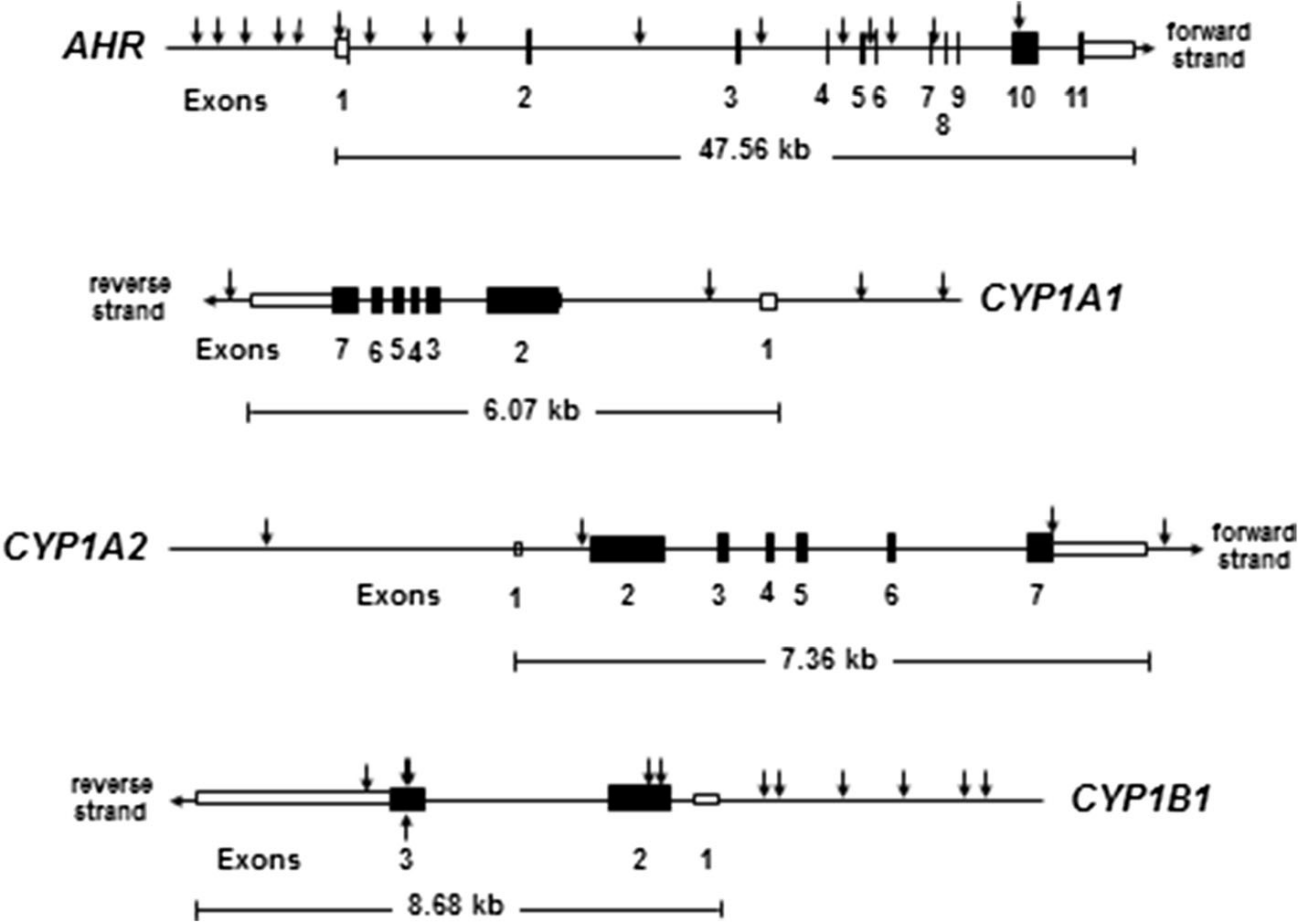
Diagram of locations of all tag-SNPs and other SNPs in the four genes chosen for study. The *AHR* gene, having 11 exons, is located on chromosome 7p15, and the transcribed region spans 47.53 kb; 16 tag-SNPs inside or near the gene were selected. *CYP1A1* (spanning 6.07 kb) and *CYP1A2* (7.36 kb) on chromosome 15q24.1 each have seven exons and are arranged in tandem, head-to-head, with a 23.3-kb bidirectional promoter between them; four tag-SNPs inside and near each gene were chosen. Human *CYP1B1* on Chr 2p22.2 has three exons and spans 8.68 kb; 12 tag-SNPs inside and near the gene were selected. Note that three SNPs (#2, 3 and 4) are located in *CYP1B1* exon 3, within four nucleotides of one another. *Closed rectangles* of the exons denote the translated region and *open rectangles* the 5′- and 3′-untranslated regions. Whereas translation of *AHR* is initiated at the 3′ end of exon 1, all three *CYP1* genes have noncoding first exons. Note that *CYP1A1* and *CYP1B1* are on reverse strand of gDNA, meaning that the chromosomal location of each SNP for these two genes in the Genome Assembly is numbered from the 3′- to 5′-end, whereas *AHR* and *CYP1A2* are on the positive strand and therefore each SNP for these two genes is numbered from the 5′- to 3′-end.

The four genes studied, with *arrows* displaying locations of each selected tag-SNP, are illustrated in Fig. 1. It is noteworthy that two of the four genes are on the reverse strand; by convention, all marker alleles were converted to the positive strand. Moreover, *CYP1A1* lies 5′-ward of *CYP1A2*, with the two genes situated head-to-head and a 23.3-kb bidirectional promoter between them; thus, two tag-SNPs located 5′-ward of *CYP1A1* and one tag-SNP 5′-ward of *CYP1A2* exon 1 are located within the bidirectional promoter—which has well-known regulatory elements, i.e., AHR-binding sites [6].

Table 2 lists the 36 selected tag-SNPs, dbSNP rs numbers, chromosomal positions, locations in or near each gene, ancestral alleles (i.e., the “phylogenetic root” based on sequence alignment of multiple (*N* = 6) primates), reference/alternative alleles, alternative allele frequencies [q(alt)] in Caucasian (EUR) samples from the 1000 Genomes Project, q(alt) in African (AFR) samples from the 1000 Genomes Project, and q(alt) found in the cohort studied (HR and HS combined). We used Genome Assembly GRCh38.p2 (Annotation Release 107) for this analysis; data were calculated from the genomic sequences of Ensembl transcripts AHR-002 (ENST00000242057), CYP1A1-001 (ENST00000379727), CYP1A2-001 (ENST00000343932), and CYP1B1-001 (ENST00000610745).

**Table 2:** List of tag-SNPs, chromosomal location, SNP identifier number, chromosomal position, location in or near gene, ancestral gene, reference/alternative alleles, alternative allele frequency q(alt) in Caucasian (EUR) and African (AFR) populations from the 1000 Genomes Project, and q(alt) in cohort studied

SNP #	Gene	Chr	SNP ID	Chromosomal position^a^	Location in or near the gene^b^	Ancestral allele^c^	Reference /alternative allele	q(alt) EUR	q(alt) AFR	q(alt) in present cohort
1	*AHR*	7	rs62444550	17290494	−8128C > A, promoter	A	C/A	0.4264	0.6407	0.4265
2	*AHR*	7	rs77821156	17291826	−6796A > G, promoter	A	A/G	0.0815	0.0045	0.0900
3	*AHR*	7	rs10250822	17293365	−5257 T > C, promoter	T	T/C	0.2217	0.0908	0.2370
4	*AHR*	7	rs4719497	17295275	−3347 T > C, promoter	T	T/C	0.1243	0.0083	0.1374
5	*AHR*	7	rs3757824	17296411	−2211 T > C, promoter	T	T/C	0.2127	0.1490	0.1706
6	*AHR*	7	rs7796976	17298806	+185G > A, 5′-UTR, exon 1	G	G/A	0.2217	0.0840	0.00
7	*AHR*	7	rs713150	17300533	+1912C > G, intron 1	C	C/G	0.2177	0.1354	0.2322
8	*AHR*	7	rs17722841	17303970	+5349G > A, intron 1	G	G/A	0.1690	0.0061	0.1919
9	*AHR*	7	rs2282885	17305990	+7369A > G, intron 1	A	A/G	0.3956	0.0189	0.3697
10	*AHR*	7	rs2282883	17316723	+18102C > T, intron 2	T	C/T	0.3469	0.6293	0.3720
11	*AHR*	7	rs4236290	17323944	+253233 T > C, intron 3	T	T/C	0.1064	0.0061	0.1114
12	*AHR*	7	rs2158041	17328796	+30175C > T, intron 4	C	C/T	0.2187	0.0340	0.2322
13	*AHR*	7	rs3802082	17330557	+31936A > T, intron 5	A	A/T	0.1769	0.1513	0.1374
14	*AHR*	7	rs7811989	17331739	+33118G > A, intron 6	G	G/A	0.2396	0.1135	0.2749
15	*AHR*	7	rs2074113	17334147	+35526G > T, intron 7	G	G/T	0.1043	0.1520	0.0711
16	*AHR*	7	rs2066853	17339486	+40865G > A, exon 10, R554K	A	G/A	0.1083	0.4622	0.0995
1	*CYP1A1*	15 (rev)	rs4646903	74719300	CYP1A1*2A, *Msp* I RFLP Site, +6311A > G, downstream 3′	A	A/G	0.1074	0.2345	0.1066
2	*CYP1A1*	15 (rev)	rs2606345	74724835	+776A > C, intron 1	C	A/C	0.3370	0.9501	0.3768
3	*CYP1A1*	15 (rev)	rs3826042	74726564	−954C > T, promoter	C	C/T	0.0417	0.0053	0.0498
4	*CYP1A1*	15 (rev)	rs7495708	74727502	−1892 T > C, promoter	C	T/C	0.1541	0.5696	0.1919
1	*CYP1A2*	15	rs2069514	74745879	CYP1A2*1C, −2965G > A, promoter	G	G/A	0.0199	0.3132	0.0616
2	*CYP1A2*	15	rs762551	74749576	CYP1A2*1 F, +733A > C, intron 1	A	A/C	0.3201	0.4380	0.2796
3	*CYP1A2*	15	rs2470890	74755085	CYP1A2*1B, +6242 T > C, exon 7, N516N (syn)	C	T/C	0.4036	0.9720	0.3981
4	*CYP1A2*	15	rs17861162	74756412	+7569C > G, downstream 3′	C	C/G	0.0626	0.1604	0.0592
1	*CYP1B1*	2 (rev)	rs162562	38070372	+5810 T > G, 3′UTR	T	T/G	0.2187	0.6694	0.2701
2	*CYP1B1*	2 (rev)	rs1800440	38070996	CYP1B1*4, +5186 T > C, exon 3, N453S	T	T/C	0.1958	0.0068	0.1588
3	*CYP1B1*	2 (rev)	rs1056837	38071007	+5175G > A, exon 3, D449D (syn)	G	G/A	0.3976	0.7988	0.4692
4	*CYP1B1*	2 (rev)	rs1056836	38071060	CYP1B1*3, +5122C > G, exon 3, L432V	C	C/G	0.3976	0.8169	0.4716
5	*CYP1B1*	2 (rev)	rs1056827	38075034	CYP1B1*2, +1148C > A, exon 2, A119S	A	C/A	0.2892	0.5234	0.2962
6	*CYP1B1*	2 (rev)	rs10012	38075247	CYP1B1*2, +935G > C, exon 2, R48G	C	G/C	0.2903	0.5741	0.2986
7	*CYP1B1*	2 (rev)	rs162558	38076937	−756 T > C, promoter	T	T/C	0.1720	0.1846	0.2085
8	*CYP1B1*	2 (rev)	rs2855655	38077346	−1165C > T, promoter	C	C/T	0.3658	0.7345	0.4431
9	*CYP1B1*	2 (rev)	rs162557	38078309	−2128G > A, promoter	G	G/A	0.2376	0.2224	0.2536
10	*CYP1B1*	2 (rev)	rs162556	38079312	−3131A > G, promoter	A	A/G	0.5000	0.0348	0.4526
11	*CYP1B1*	2 (rev)	rs162555	38080367	−4186 T > C, promoter	T	T/C	0.1750	0.1664	0.2014
12	*CYP1B1*	2 (rev)	rs10175368	38080719	−4538C > T, promoter	C	C/T	0.2853	0.0613	0.2559

*Chr* chromosome, *rev* reverse strand, *syn* synonymous, *q(alt)* frequency of alternative allele

^a^Genome Assembly GRCh38.p2 (Annotation Release 107)

^b^Calculated from the genomic sequences of Ensembl transcripts AHR-002 (ENST00000242057), CYP1A1-001(ENST00000379727), CYP1A2-001 (ENST00000343932), and CYP1B1-001 (ENST00000610745)

^c^Ancestral allele refers to the “phylogenetic root” based on sequence alignment of multiple (*N* = 6) primates, as reported in dbSNP

### Single-marker association study (allele test)

The entire cohort (HS and HR combined, *N* = 211) included 196 self-identified Caucasian-Americans, 14 African-Americans, and 1 Latino (Table 1). Using the chi-square test for allele-frequency difference of each tag-SNP between HS and HR samples (Table 3), we found three *P* values significant at the *P* < 0.05 level; however, these associations did not retain statistical significance in the 100,000 permutations test. This is a very common observation and often not appreciated—when comparing standard statistical tests with a permutation test that is mandatory for association studies with regard to multiple markers throughout all chromosomes [4].

**Table 3:** Comparison of allele frequencies (additive model), as tested by chi-square analysis and permutations test, of individual tag-SNPs, HS vs. HR samples, entire cohort (*N* = 211)

Gene	Tag-SNP #	SNP ID	Asso-ciated allele^a^	Cases (HS) allele frequencies	Controls (HR) allele frequencies	*P* value (chi-square analysis)	*P* value, following 100 K permutations
*AHR*	1	rs62444550	A	0.433	0.419	0.7741	1.00
*AHR*	2	rs77821156	A	0.929	0.889	0.1553	0.7627
*AHR*	3	rs10250822	C	0.272	0.197	0.0693	0.4745
*AHR*	4	rs4719497	C	0.174	0.096	0.0200^*^	0.1727
*AHR*	5	rs3757824	T	0.862	0.793	0.0613	0.4203
*AHR*	7	rs713150	G	0.263	0.197	0.1068	0.6212
*AHR*	8	rs17722841	G	0.817	0.798	0.6212	0.9998
*AHR*	9	rs2282885	A	0.665	0.591	0.1147	0.6360
*AHR*	10	rs2282883	T	0.397	0.343	0.2531	0.9128
*AHR*	11	rs4236290	T	0.902	0.874	0.3607	0.9774
*AHR*	12	rs2158041	T	0.250	0.212	0.3578	0.9742
*AHR*	13	rs3802082	A	0.884	0.838	0.1751	0.8056
*AHR*	14	rs7811989	A	0.295	0.253	0.3335	0.9620
*AHR*	15	rs2074113	G	0.942	0.914	0.2670	0.9201
*AHR*	16	rs2066853	A	0.107	0.091	0.5783	0.9996
*CYP1A1*	1	rs4646903	G	0.125	0.086	0.1936	0.7207
*CYP1A1*	2	rs2606345	C	0.397	0.354	0.3543	0.9355
*CYP1A1*	3	rs3826042	C	0.960	0.939	0.3355	0.9121
*CYP1A1*	4	rs7495708	C	0.205	0.177	0.4567	0.9711
*CYP1A2*	1	rs2069514	A	0.089	0.030	0.0119 ^*^	0.0896
*CYP1A2*	2	rs762551	A	0.723	0.717	0.8902	1.0000
*CYP1A2*	3	rs2470890	C	0.438	0.354	0.0787	0.4071
*CYP1A2*	4	rs17861162	G	0.076	0.040	0.1233	0.5885
*CYP1B1*	1	rs162562	G	0.317	0.217	0.0212 ^*^	0.1262
*CYP1B1*	2	rs1800440	T	0.862	0.818	0.2232	0.7441
*CYP1B1*	3	rs1056837	A	0.478	0.460	0.7103	0.9998
*CYP1B1*	4	rs1056836	C	0.482	0.460	0.6433	0.9979
*CYP1B1*	5	rs1056827	A	0.317	0.273	0.3206	0.8683
*CYP1B1*	6	rs10012	C	0.326	0.268	0.1922	0.6692
*CYP1B1*	7	rs162558	T	0.821	0.758	0.1071	0.4914
*CYP1B1*	8	rs2855655	C	0.562	0.551	0.8045	1.0000
*CYP1B1*	9	rs162557	A	0.272	0.232	0.3459	0.9051
*CYP1B1*	10	rs162556	A	0.567	0.525	0.3903	0.9346
*CYP1B1*	11	rs162555	T	0.826	0.768	0.1367	0.5529
*CYP1B1*	12	rs10175368	C	0.746	0.742	0.9417	1.0000

^*^Statistically significant (*P* < 0.05)

^a^Associated allele, as selected by the HaploView program

Case-control association tests in Caucasians (*N* = 196) were achieved by removing the 1 Latino and 14 African-American subjects. Table 4 displays the HaploView analysis—examining individual markers or SNPs. From chi-square analysis, there is only one SNP that appears to show a significant *P* value in the *AHR* gene: rs4719497 (*P* = 0.0134). All other SNPs have *P* values >0.05. However, following permutation testing, rs4719497 did not retain significance (*P* = 0.1048), indicating that this SNP did not survive the mandatory correction for multi-testing.

**Table 4:** Comparison of allele frequencies (additive model), as tested by chi-square analysis and permutations test, of individual tag-SNPs, HS vs. HR Caucasian-only sample (*N* = 196)

Gene	Tag-SNP #	SNP ID	Asso-ciated allele^a^	Cases (HS) allele frequencies	Controls (HR) allele frequencies	*P* value (chi-square analysis)	*P* value, following 100 K permutations
*AHR*	1	rs62444550	A	0.926	0.884	0.1598	0.741
*AHR*	2	AHR10SNP	A	0.431	0.416	0.7654	1.00
*AHR*	3	rs10250822	C	0.282	0.205	0.0768	0.4705
*AHR*	4	rs4719497	C	0.188	0.100	0.0134 ^*^	0.1048
*AHR*	5	rs3757824	T	0.856	0.795	0.1069	0.6213
*AHR*	7	rs713150	G	0.272	0.205	0.1204	0.6554
*AHR*	8	rs17722841	G	0.797	0.795	0.9551	1.00
*AHR*	9	rs2282885	A	0.629	0.579	0.3139	0.9577
*AHR*	10	rs2282883	T	0.386	0.337	0.3102	0.9496
*AHR*	11	rs4236290	T	0.896	0.868	0.3958	0.9802
*AHR*	12	rs2158041	T	0.267	0.221	0.287	0.9119
*AHR*	13	rs3802082	A	0.876	0.842	0.3308	0.9603
*AHR*	14	rs7811989	A	0.302	0.263	0.3939	0.9776
*AHR*	15	rs2074113	G	0.941	0.921	0.4452	0.992
*AHR*	16	rs2066853	A	0.084	0.079	0.8506	1.00
*CYP1A1*	1	rs4646903	G	0.104	0.084	0.5039	0.9866
*CYP1A1*	2	rs2606345	C	0.351	0.337	0.7604	0.9999
*CYP1A1*	3	rs3826042	C	0.955	0.937	0.4136	0.9446
*CYP1A1*	4	rs7495708	C	0.158	0.153	0.8745	1.00
*CYP1A2*	1	rs2069514	A	0.035	0.016	0.2365	0.8158
*CYP1A2*	2	rs762551	C	0.292	0.279	0.7736	1.00
*CYP1A2*	3	rs2470890	C	0.386	0.332	0.2606	0.8355
*CYP1A2*	4	rs17861162	G	0.064	0.037	0.216	0.7605
*CYP1B1*	1	rs162562	G	0.282	0.221	0.1639	0.6189
*CYP1B1*	2	rs1800440	T	0.856	0.821	0.3404	0.8777
*CYP1B1*	3	rs1056837	G	0.545	0.542	0.9612	1.00
*CYP1B1*	4	rs1056836	C	0.460	0.458	0.9604	1.00
*CYP1B1*	5	rs1056827	A	0.297	0.268	0.5298	0.9853
*CYP1B1*	6	rs10012	C	0.302	0.263	0.3939	0.9263
*CYP1B1*	7	rs162558	T	0.812	0.763	0.238	0.751
*CYP1B1*	8	rs2855655	C	0.589	0.547	0.4043	0.9302
*CYP1B1*	9	rs162557	A	0.277	0.242	0.4283	0.9473
*CYP1B1*	10	rs162556	A	0.540	0.516	0.6369	0.9954
*CYP1B1*	11	rs162555	T	0.817	0.774	0.2895	0.8232
*CYP1B1*	12	rs10175368	T	0.277	0.258	0.6658	0.9985

^*^ Statistically significant (*P* < 0.05)

^a^Associated allele, as selected by the HaploView program

### Haplotype association study

**Fig. 2:**
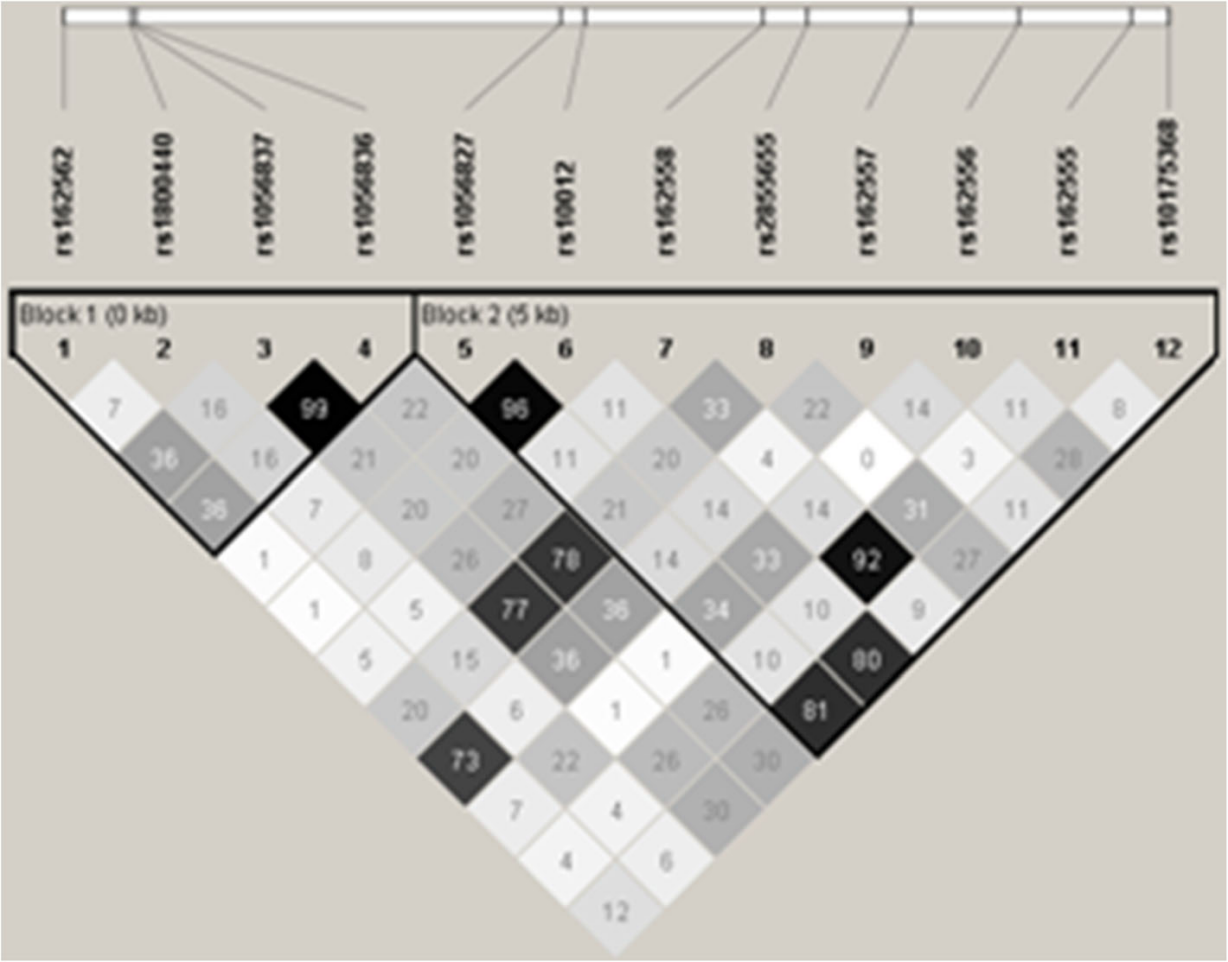
LD heat map of *CYP1B1* SNPs from the entire cohort (*N* = 211), using *r* ^2^ (correlation between pair of loci) as a measure of LD. Haploblock 2—which we had found initially as apparently the only haplotype (ACTTGATC) significantly associated with HNSCC risk in smokers—contains two SNPs in the 5′ half of exon 2 plus six SNPs in the 5′-flanking region (reverse strand), extending as far 5′-ward as 4538 bp upstream of the transcription initiation start-site. *Shading* denotes the following: *white* (*r* ^2^ = 0), black (*r* ^2^ = 1), and shades of *gray* (0 < *r* ^2^ < 1), with deepening *gray colors* depicting increasing *r* ^2^ values (correlation) between SNP pairs. *Numbers* in each *square* indicate percentage of correlation (*r* ^2^ × 100).

Figure 2 shows the LD heat map for *CYP1B1* SNPs, using *r* ^2^ (correlation between pairs of loci) as a measure of linkage disequilibrium. Haploblocks 1 (tag-SNPs #1, 2, 3, and 4) and 2 (tag-SNPs #5, 6, 7, 8, 9, 10, 11, and 12) were determined by the program HaploView because they showed the best continuous solid spine of LDs. We defined haploblocks by applying this nonstringent criterion—since no strong correlations were expected between tag-SNP pairs, i.e., by definition, tag-SNPs are selected because they should capture as much information as possible about independent regions of the gene. However, some of the SNPs selected for this study are in very close proximity to one other and did show substantially strong correlations (depicted as *darkest squares* in the heat map, e.g., SNPs #5 and 6). We included these SNPs in our study because of functional relevance in cancer studies or known to alter enzyme activity levels. LD heat maps and haploblocks were similarly generated for the other three genes studied and were unremarkable (*data not shown*).

Of the 38 haplotypes inferred in the six defined haploblocks among our four genes in the entire cohort (Table 5, *N* = 211), six were statistically significant (*P* < 0.05) by chi-square analysis. After 100,000 permutations, however, only one *CYP1B1* haplotype in haploblock 2 (ACTTGATC) retained a statistically significant (*P* = 0.0042) association with HNSCC risk in smokers.

**Table 5:** Comparison of haplotype frequencies (additive model), as tested by chi-square analysis and permutations test, HS vs. HR samples, entire cohort (*N* = 211)

Haploblock and inferred haplotypes	Haplotype total frequency	Cases (HS) frequencies	Controls (HR) frequencies	*P* value (chi-square analysis)	*P* value, following 100 K permutations
*AHR* Block 1					
ACTTTC	0.479	0.487	0.470	0.7288	1.00
AATTCC	0.171	0.138	0.207	0.0613	0.5326
AACCTG	0.133	0.165	0.096	0.0365 ^*^	0.3665
AACTTG	0.097	0.094	0.101	0.8016	1.0000
GCTTTC	0.090	0.071	0.111	0.1553	0.8236
AATTTC	0.024	0.031	0.015	0.2781	0.9680
*AHR* Block 2					
GACTCAGGG	0.243	0.249	0.237	0.7678	1.00
AGCTCAGGG	0.187	0.174	0.201	0.464	1.00
GATTTAAGG	0.152	0.175	0.126	0.17	0.8445
GGCTCAGGG	0.119	0.108	0.132	0.4426	1.0000
GATCTAAGG	0.075	0.066	0.085	0.4563	1.0000
GATTCTGTA	0.071	0.058	0.086	0.267	0.9607
GGCTCTGGG	0.061	0.049	0.075	0.2578	0.9456
GATCCAAGG	0.033	0.031	0.035	0.8143	1.00
GATTCAGGA	0.026	0.045	0.005	0.0109 ^*^	0.0786
GACTCAAGG	0.012	0.018	0.005	0.225	0.9396
*CYP1A1*					
AACT	0.623	0.603	0.646	0.3543	0.9613
ACCT	0.185	0.192	0.177	0.6882	0.9998
GCCC	0.107	0.125	0.086	0.1936	0.7935
ACTC	0.050	0.040	0.061	0.3355	0.9493
ACCC	0.036	0.040	0.030	0.5845	0.9975
*CYP1A2*					
GAT	0.602	0.562	0.646	0.0787	0.4736
GCC	0.280	0.277	0.283	0.8902	1.00
AAC	0.062	0.089	0.030	0.0119 ^*^	0.1239
GAC	0.057	0.071	0.040	0.1697	0.7649
*CYP1B1* Block 1					
TTGG	0.360	0.364	0.357	0.8781	1.00
GTAC	0.261	0.301	0.215	0.0444 ^*^	0.2237
TTAC	0.208	0.176	0.244	0.086	0.5013
TCGG	0.159	0.138	0.182	0.2232	0.8763
*CYP1B1* Block 2					
ACTCGATT	0.256	0.254	0.258	0.9417	1.00
CGTCGGTC	0.251	0.237	0.268	0.4627	1.00
CGCTGACC	0.185	0.156	0.217	0.1078	0.6369
CGTTAGTC	0.182	0.174	0.192	0.6369	1.00
CGTCAATC	0.037	0.057	0.015	0.0248	0.1204
ACTTGATC	0.028	0.052	0.000	0.0011 ^**^	0.0042 ^**^
CGTTAATC	0.020	0.024	0.015	0.5256	1.00
CGCTAGCC	0.012	0.013	0.010	0.7551	1.00
ACTCGATC	0.010	0.010	0.010	0.9756	1.00

*AHR Block 1* SNPs 1, 2, 3, 4, 5, 7, *AHR Block 2* SNPs 8, 9, 10, 11, 12, 13, 14, 15, 16, *CYP1A1* SNPs 1, 2, 3, 4, *CYP1A2* SNPs 1, 2, 3, *CYP1B1 Block 1* SNPs 1, 2, 3, 4, *CYP1B1 Block 2* SNPs 5, 6, 7, 8, 9, 10, 11, 12

^*^Statistically significant (*P* < 0.05)

^**^Statistically significant (*P* < 0.005)

The same analysis was applied to the Caucasian-only sample (*N* = 196) and the results are shown in Table 6. Only two haplotypes revealed significant *P* values: one in *AHR* Block 1 (*P* = 0.0259) and one in *CYP1B1* Block 2 (*P* = 0.0392). Following correction for multi-testing (100K permutations), however, the appearance of statistical significance was lost: *P* = 0.142 and 0.1666, respectively. Note that the inferred haplotypes, and order of their ranking by frequency (*left-most columns*), differs between Table 5 (entire cohort) and Table 6 (Caucasian-only sample).

**Table 6:** Comparison of haplotype frequencies (additive model), as tested by chi-square analysis and permutations test, HS vs. HR Caucasian-only sample (*N* = 196)

Haploblock and inferred haplotypes	Haplotype total frequency	Cases (HS) frequencies	Controls (HR) frequencies	*P* value (chi-square analysis)	*P* value, following 100 K permutations
*AHR* Block 1					
ACTTTC	0.477	0.485	0.468	0.7405	1.00
AATTCC	0.173	0.144	0.205	0.1069	0.6743
AACCTG	0.14	0.178	0.100	0.0259 ^*^	0.142
AACTTG	0.097	0.089	0.105	0.589	1.00
GCTTTC	0.094	0.074	0.116	0.1598	0.8367
AATTTC	0.01	0.015	0.005	0.3454	1.00
*AHR* Block 2					
GACTCAGGG	0.231	0.232	0.231	0.9865	1.00
AGCTCAGGG	0.198	0.193	0.205	0.7632	1.00
GATTTAAGG	0.159	0.184	0.132	0.16	0.8367
GGCTCAGGG	0.128	0.119	0.137	0.5925	1.00
GATCTAAGG	0.081	0.073	0.089	0.5716	1.00
GATTCTGTA	0.069	0.059	0.079	0.4451	1.00
GGCTCTGGG	0.066	0.054	0.079	0.3323	1.00
GATCCAAGG	0.033	0.030	0.037	0.6932	1.00
GATTCAGGA	0.01	0.020	0.000	0.0512	0.3157
*CYP1A1*					
AACT	0.656	0.649	0.663	0.7604	1.00
ACCT	0.189	0.193	0.184	0.8228	1.00
GCCC	0.094	0.104	0.084	0.5039	0.9829
ACTC	0.054	0.045	0.063	0.4136	0.9422
*CYP1A2*					
GAT	0.64	0.614	0.668	0.2606	0.8454
GCC	0.286	0.292	0.279	0.7736	1.00
GAC	0.048	0.059	0.037	0.2985	0.888
AAC	0.026	0.035	0.016	0.2365	0.8278
*CYP1B1* Block 1					
TTGG	0.377	0.390	0.362	0.5704	1.00
GTAC	0.249	0.276	0.220	0.1999	0.8872
TTAC	0.207	0.179	0.238	0.1535	0.8046
TCGG	0.161	0.144	0.179	0.3404	1.00
*CYP1B1* Block 2					
ACTCGATT	0.268	0.277	0.258	0.6658	1.00
CGTCGGTC	0.258	0.248	0.268	0.6364	1.00
CGTTAGTC	0.194	0.188	0.200	0.7667	1.00
CGCTGACC	0.186	0.163	0.210	0.2309	0.9115
CGTCAATC	0.036	0.054	0.016	0.0392 ^*^	0.1666
CGTTAATC	0.015	0.015	0.016	0.9386	1.00
CGCTAGCC	0.013	0.015	0.011	0.7029	1.00

*AHR Block 1* SNPs 1, 2, 3, 4, 5, 7, *AHR Block 2* SNPs 8, 9, 10, 11, 12, 13, 14, 15, 16, *CYP1A1* SNPs 1, 2, 3, 4, *CYP1A2* SNPs 1, 2, 3, *CYP1B1 Block 1* SNPs 1, 2, 3, 4, *CYP1B1 Block 2* SNPs 5, 6, 7, 8, 9, 10, 11, 12

^*^Statistically significant (*P* < 0.05)

### Further analysis of *CYP1B1* haplotype ACTTGATC

In our initial approach, all HS and HR subjects (*N* = 211, i.e., 422 chromosomes, including one self-identified Latino) were included in the same association study of haplotype ACTTGATC with HNSCC risk among smokers, irrespective of racial origin (Table 5); and it was initially exciting to find the ACTTGATC haplotype (apparently) statistically significantly associated (*P* = 0.0042) with cigarette smoking-induced risk of HNSCC. When the Latino subject was excluded from the analysis, leaving only African-American and Caucasian-Americans (Table 7), Fisher’s exact test indicated a significant difference in the frequency of this haplotype between HS and HR (*P* = 0.0011).

**Table 7:** Detailed studies of chromosomal frequencies of *CYP1B1* haplotype ACTTGATC in haploblock 2

Caucasian-Americans and African-Americans in present cohort, combined (Latino-American excluded)			
	ACTTGATC^*^	Other haplotypes	Total chromosomes
HS	11	213	224
HR	0	196	196
Total	11	409	420

^*^ Statistically significant (*P* = 0.0011) by Fisher’s exact test, comparing HS with HR

However, when individuals carrying this haplotype were identified by race (Table 8), it became apparent that the haplotype was tracking extensively with African origin, rather than HNSCC risk; in a total of 112 HS subjects (224 chromosomes and excluding the one Latino), this haplotype appeared 11 times—8 times in heterozygous state in African-Americans and 3 times in heterozygous state among Caucasian-Americans. This distribution is disproportionately larger for African-Americans, considering we had recruited only 11 African-American HS vs. 101 Caucasian-American HS patients. The Fisher exact test demonstrated a significant difference (*P* < 0.0001) in this haplotype frequency between African-American and Caucasian-American HS chromosomes (Table 8).

**Table 8:** Detailed studies of chromosomal frequencies of *CYP1B1* haplotype ACTTGATC in haploblock 2

Ethnicity (self-identified)	ACTTGATC^*^		Other haplotypes		Total chromosomes
	HS	HR	HS	HR	
Caucasian-American (CAU)	3	0	199	190	392
African-American (AFR)	8	0	14	6	28
Latino-American	0	0	0	2	2
Total	11	0	213	198	422

^*^Statistically significant (*P* < 0.0001) by Fisher’s exact test (two-tailed), comparing AFR with CAU (HS patients)

From a total of 28 African-American chromosomes of both HS and HR studied (Table 9), 8 carried the ACTTGATC haplotype (allele frequency = 0.286). However, when we compared this haplotype and HNSCC risk in African-Americans, no statistically significant conclusion could be reached (*P* = 0.141), perhaps because our study is under-powered due to small sample sizes—especially a control HR group comprising only six chromosomes.

**Table 9:** Detailed studies of chromosomal frequencies of *CYP1B1* haplotype ACTTGATC in haploblock 2

African-Americans in present cohort			
	ACTTGATC^*^	Other haplotypes	Total chromosomes
HS	8	14	22
HR	0	6	6
Total	8	20	28

^*^Not significantly different (*P* = 0.141) by Fisher’s exact test, comparing HS with HR

To investigate the possibility that haplotype ACTTGATC in our African-American cohort simply represented a high-frequency haplotype among Africans, we turned to the 1000 Genomes Project and searched for average frequencies of each SNP within *CYP1B1* haploblock 2 among African populations; we inferred the best reconstructed haplotypes and estimated their frequencies (Additional file 2: Table S1). Interestingly, haplotype ACTTGATC was found to be the most common (ranked #1)—among the sum of all African genomes sequenced to date—with a frequency of 0.356.

Regarding the majority of our HNSCC cohort who were of self-identified Caucasian descent (*N* = 196, i.e., 392 chromosomes), we failed to find an association (*P* = 0.249) between haplotype ACTTGATC and HNSCC risk (Table 10). Using data extracted from the 1000 Genomes Project for European populations (Additional file 2: Table S1) and analyzing it similarly to what had been done for African populations, we found that haplotype ACTTGATC has a frequency of 0.003—ranking #9 among inferred haplotypes for Europeans.

**Table 10:** Detailed studies of chromosomal frequencies of *CYP1B1* haplotype ACTTGATC in haploblock 2

Caucasian-only sample			
	ACTTGATC^*^	Other haplotypes	Total chromosomes
HS	3	199	202
HR	0	190	190
Total	3	389	392

^*^Not significantly different (*P* = 0.249) by Fisher’s exact test, comparing HS with HR

Taking into account the significant difference (*P* < 0.0001) in frequency of haplotype ACTTGATC between European and African populations represented in the 1000 Genomes Project (Table 11), and the apparent lack of significant association between this haplotype and HNSCC risk when African-Americans (Table 9) and Caucasian-Americans (Table 10) were analyzed separately, we are able confidently to conclude that the association found in our initial analysis was spurious and caused by *population stratification*—a common artifact that can lead many statisticians to reporting a false positive (type I error) as a true positive.

**Table 11:** Detailed studies of chromosomal frequencies of *CYP1B1* haplotype ACTTGATC in haploblock 2

Caucasians (EUR) and Africans (AFR) in The 1000 Genomes Project				
	ACTTGATC^*^	Other haplotypes	Total chromosomes	Haplotype frequency
EUR	3	1003	1006	0.003
AFR	471	851	1332	0.356
Total	474	1854	2328	

^*^Statistically significant (*P* < 0.0001) by Fisher’s exact test, comparing EUR with AFR

## Discussion

The present study represents a candidate-gene analysis, using the extreme discordant phenotype (EDP) method, attempting to determine a genotype-phenotype association—in which the trait being studied is HNSCC among cigarette smokers. The four candidate genes included *AHR*, *CYP1A1*, *CYP1A2*, and *CYP1B1*. Based on innumerable laboratory animal studies, these four genes were selected because they encode three CYP1 enzymes, plus AHR which regulates the levels of these enzymes. This gene battery [27] reflects one of the earliest responses to cigarette smoke as an environmental signal. Moreover, HNSCC is most likely to develop as the result of “direct-contact” of head-and-neck epithelial cells with chronic irritants in cigarette smoke. For example, if any nonsmoker were to smoke even one cigarette—these three *CYP1* genes would become up-regulated by AHR (to genetically varying degrees) within a few hours; without further “stimulation” (i.e., no more cigarettes), AHR-regulated *CYP1* genes would quickly return to baseline. In laboratory animals, there is ample evidence that these four genes play an “up-front” role in PAH-induced cancer [5, 27, 38].

### Statistical power of EDP analysis

An accurate power calculation of EDP design—based on exposure level [EDP design 2 of the [46] article [to a known risk factor (e.g., cigarette smoking)] ﻿—would require quantification of several features: (a) phenotypic variance attributable to the environmental exposure, (b) truncation selection areas (e.g., 1–40 cigarette pack-years for HS cases, >80 cigarette pack-years for HR controls), and (c) disease prevalence. Given that these values are not well known, it would be very difficult to provide an accurate power calculation.

However, if we can roughly assume that cigarette smoking explains >20% variance in liability of HNSCC that the selection areas (1–40 vs. >80 cigarette pack-years) represent the bottom and the top 10% exposure groups, respectively, and that the prevalence of HNSCC in all smokers is ~1%, then our EDP design would have similar power to a normal case/control study comprising 500~600 samples (Fig. 9 of the [46] article).

### Previous genotype-phenotype association studies: HNSCC and our four candidate genes

We found no studies in the literature concerning *AHR* polymorphisms associated with HNSCC. With regard to *CYP1* polymorphisms and HNSCC risk, six of the seemingly most relevant studies are detailed below.

(**a**) The Leu-432 allele of *CYP1B1* has been associated with a greater frequency [odds-ratio (O.R.) >4] of *TP53* gene mutations and HNSCC in cigarette smokers [16]; however, this “greater risk” is difficult to reconcile experimentally—given the fact that the Leu-432 mutant was shown in an *E. coli* expression assay in vitro to metabolize PAHs only 1.2- to 1.5-fold better than the Val-432 variant [42]. (**b**) Four nonsynonymous coding SNPs (p.R48G, p.A119S, p.L432V, and p.N453S) were studied in 150 cases of HNSCC and 150 controls [43]; when the four SNPs were analyzed as a haplotype, amino acid changes p.A48G and p.A119S exhibited complete LD in all cases and controls, and significant differences (*P* < 0.05) were reported in the two haplotypes—GTCA and GTGA—as being associated with increased risk of HNSCC. (**c**) Another study, comparing 312 HNSCC cases and 300 noncancer controls, looked at the impact of 22 sequence variations in *CYP1A1*, *CYP1B1*, *CYP2E1*, *GSTM1*, and six other genes encoding either xenobiotic-metabolizing or DNA repair enzymes [13]; using logit regression and a Bayesian version of logit regression, authors found significant (*P* < 0.05) associations of *CYP1B1* p.L432V and *CYP2E1* −70G > T (O.R. 10.84; 95% CI, 1.64–71.53), as well as *CYP1B1* p.L432V and *GSTM1(0/0)* null/null (OR 11.79; 95% CI, 2.18–63.77) with HNSCC risk. (**d**) In another study of 153 HNSCC cases and 145 controls with no current or previous diagnosis of cancer [37], authors examined *CYP1A1*, *CYP1A2*, *CYP2E1*, *GSTM1*, and *GSTT1* SNPs as risk factors in HNSCC; a significant difference (*P* < 0.001) was detected for tobacco and alcohol consumption between cases and controls; moreover, the CYP1A2*1D (OR 16.24) variant and *GSTM1(0/0)* null alleles (OR 0.02) conferred increased risk of HNSCC, and alcohol consumption in HNSCC patients was associated with the CYP2E1*5B variant allele (*P* < 0.0001, OR 190.6). (**e**) Performing meta-analysis of six published case-control studies of HNSCC—which included some studies that had concluded no significant risk—authors [41] found that the *CYP1B1* p.L432V polymorphism was significantly related with HNSCC risk (OR = 1.13, 95% CI = 1.03–1.25, *P* = 0.014), whereas no significant association between *CYP1B1* p.N453S polymorphism and HNSCC risk was found. (**f**) Finally, a case-control study of 750 HNSCC patients and equal number of healthy controls investigated the association of polymorphisms in *CYP1A1*, *CYP1B1*, *CYP2E1*, and *GSTM1* with HNSCC [24]; cases having variant haplotypes of both CYP1A1*2A and CYP1A1*2C or CYP1B1*2 and CYP1B1*3 or CYP2E1*5B and CYP2E1*6 were at significant (*P* < 0.05) risk of developing HNSCC; statistical analysis revealed a more than multiplicative interaction between combinations of these variant *CYP* genotypes plus the *GSTM1(0/0)* null genotype and between variant genotypes and tobacco smoking or chewing or alcohol consumption—indicating these genes contribute a “modest risk factor” for developing HNSCC via gene-gene and gene-environment interactions. To learn more about each *CYP* allele described above, please refer to http://www.cypalleles.ki.se/

### What sets our study apart from all previous studies?

None of the studies mentioned above, however, had distinguished between HNSCC in smokers and nonsmokers. Also, in contrast to the present study that used the EDP method of sample selection, all the studies mentioned above used, as their control population—nonsmokers, or any smoker not having cancer. Furthermore, none of these studies employed any form of multi-locus statistical testing; this is a very serious recurring problem in the medical literature with regard to studies attempting to discover associations between one or more SNPs and a multifactorial trait [4, 36], whi﻿ch is a type I (false-positive) error variously called “*P* < 0.05 studies” [36] and the “incidentalome” [18].

### A type I error (false-positive) likely resulting from population stratification

The present study, at least based on the size of our cohort, rejected all four *AHR*, *CYP1A1*, *CYP1A2*, and *CYP1B1* genes as having any statistically significantly detectable contribution to HNSCC in smokers; further, we first found a false-positive observation—that the *CYP1B1* haplotype ACTTGATC might be associated with HNSCC risk in our cohort (Table 7). Instead, this finding was realized to be extremely unlikely, due to haplotype frequency differences of more than 100-fold between African and Caucasian populations in the 1000 Genomes Project (Additional file 2: Table S1); hence, our study is an excellent teaching example of the consequences of a population stratification artifact.

### Increasing appreciation of CYP1-mediated detoxication, as well as metabolic activation

One additional consideration is that we now know from transgenic mouse studies that all three CYP1 enzymes can participate in both metabolic activation and detoxication—depending on dose, length of exposure, route of administration, and target organ specificity [5, 35, 38]. Therefore, it seems clear that virtually all previous epidemiological studies are ambiguous because such studies have considered only the “metabolic activation” component [29]. Yet, when cancer initiation occurs via “direct contact” of a carcinogen—such as cigarette smoke exposure causing HNSCC—one would presume that CYP1-mediated metabolic activation [27] would be a much more likely determinant than detoxication [35].

However, in the population studied herein, we failed to demonstrate a correlation between the genotype of *AHR* and all three *CYP1* genes and HNSCC in smokers. This does not mean that—if a much larger cohort were collected—an association between HNSCC and small-effect contribution by one or another of these four genes might not emerge. We found a lack of association because our Caucasian and African cohorts were apparently too small, especially the African population (both HS and HR).

### Environmental risk factors for HNSCC

Tobacco (smoking cigarettes, cigars or pipe, second-hand smoke, chewing tobacco, and using snuff) probably represents the single greatest risk factor for developing HNSCC. The vast majority (~85%) of HNSCC is associated with tobacco usage. Heavy and frequent alcohol consumption is the second highest risk factor. Often intertwined with tobacco and heavy alcohol usage, poor oral and dental hygiene, malnutrition, and cannabis usage may enhance HNSCC risk. Men are two- to threefold more likely than women to develop HNSCC, and patients above age 40 are at higher risk. Exposure to occupational inhalants (wood dust, paint fumes, and certain chemicals) may increase a person’s HNSCC risk. Finally, gastroesophageal-reflux disease (GERD), laryngopharyngeal-reflux disease (LPRD), and a weakened immune system can raise the risk of HNSCC. HPV is commonly seen in some types of HNSCC, as detailed below.

### Past and present concepts of the role of HPV in HNSCC

Over the past two decades, HPV-positive squamous cell carcinoma has emerged as a distinct subset of HNSCC, based on multiple lines of evidence. Such evidence for an etiologic role of HPV includes (**a**) observations of higher rate of anti-HPV antibody sero-positivity—even after adjustment for smoking—among patients with HNSCC, compared with that among cancer-free individuals [25]; (**b**) preferential occurrence of HPV-positive tumors in the oropharynx [12]; (**c**) unique demographic features, including an increased number of lifetime sexual partners and less exposure to tobacco and alcohol [11]; (**d)** lower rates of *TP53* gene mutations, in HPV-positive than HPV-negative oropharyngeal tumors [12]; and (**e**) improved survival among HPV-positive oropharynx cancers, compared to HPV-negative oropharynx carcinomas in both retrospective [2] and prospective [9] analyses of clinical trial data.

Given the clinical impact of HPV infection in oropharyngeal SCCs, clinical testing for the presence of virus in newly diagnosed tumors at this site is considered mandatory and guides therapy—including enrollment in clinical trials investigating de-intensified radiotherapy regimens [23]. Such testing may include immunohistochemical staining for p16, the overexpression of which occurs when HPV proteins E6 and E7 inactivate TP53 and RB1 proteins, thereby serving as a surrogate marker of viral infection. Alternatively, HPV-specific methods such as in situ hybridization staining of viral DNA or polymerase chain reaction (PCR)-based detection of viral DNA or RNA may be used. Many argue that p16 immunohistochemistry is sufficient for oropharyngeal carcinomas [22] and that PCR-based tests may be overly sensitive and detect latent HPV unrelated to carcinogenesis in a given tumor [21].

Despite the well-established role of HPV in oropharyngeal SCC and its attendant clinical implications, classic carcinogenic effects of tobacco and alcohol appear to abrogate the improved survival of these tumors. Specifically, HPV infection may occur after significant exposure to tobacco and/or alcohol and therefore may not be associated with a statistically different outcome than HPV-negative tumors [45]. On the basis of these mitigating effects of tobacco exposure in HPV-positive tumors, risk stratification schemes that incorporate both smoking history and tumor HPV status have been developed [2]. It should also be noted that the etiologic role and clinical impact of HPV in nonoropharyngeal SCC has not been clearly established; for example, the prevalence of virus in a study of oral cavity SCCs was low, and clinical features of patients with, vs. without, tumor-containing HPV were similar [19].

Whereas our cohort did include 15-20% oropharyngeal SCCs, we believe our results are still applicable to this subset, although we did not analyze the tumors for HPV infection. Specifically, it may be argued that—because the carcinogenic effects of tobacco appear to supersede the effects associated with HPV-driven oropharyngeal SCC, and not all oropharyngeal tumors are etiologically due to HPV—the lack of genotype-phenotype correlation that we have clearly demonstrated in this study is highly likely to hold true in the oropharynx, as well as in other head-and-neck subsites.

## Conclusions

With regard to our EDP method of rigorously selecting cohorts of *N* = 112 highly-sensitive (HS) light smokers with HNSCC and *N* = 99 highly resistant (HR) heavy smokers with no cancer, we conclude that the carefully chosen single-nucleotide variants located in and near four genes—*AHR*, *CYP1A1*, *CYP1A2*, *CYP1B1*, alone, or in combination—are not statistically significantly associated with risk of cigarette-smoking-induced HNSCC. One haploblock, ACTTGATC in the 5′ portion of *CYP1B1*, retained statistical significance after 100,000 permutations, but this was discovered to be a type I error (false positive) finding, due to spurious association by population stratification.

## Additional files

Additional file 1: Questionnaire for genetic study of head-and-neck cancer. (DOC 43 kb)
Additional file 2: Supplemental data online. (DOC 64 kb)

## Abbreviations

AHR: Aryl hydrocarbon receptor protein
*AHR*: Gene encoding aryl hydrocarbon receptor
Cig-Pk-Yr: Cigarette-pack-year (20 cigarettes smoked per day for 1 year)
*CYP1A1*: Gene encoding cytochrome P450 family 1 subfamily A member 1
CYP1A1: Name of enzyme
*CYP1A2*: Gene encoding cytochrome P450 family 1 subfamily A member 2
CYP1A2: Name of enzyme
*CYP1B1*: Gene encoding cytochrome P450 family 1 subfamily B member 1
CYP1B1: Name of enzyme
EDP: Extreme discordant phenotype method of population analysis
gDNA: Genomic DNA
HNSCC: Head-and-neck squamous cell carcinoma
HPV: Human papilloma virus
LD: Linkage disequilibrium
SNPs: Single nucleotide polymorphisms, variant alleles
