# Supplementary material for: Head-and-neck squamous cell carcinoma risk in smokers: no association detected between phenotype and AHR, CYP1A1, CYP1A2, or CYP1B1 genotype
Source: Hum Genomics. 2016 Nov 28;10:39. doi: 10.1186/s40246-016-0094-y (PMC5127090; doi:10.1186/s40246-016-0094-y)
Supplement: Additional file 1: — Questionnaire for genetic study of head-and-neck cancer. (DOC 43 kb) [file 40246_2016_94_MOESM1_ESM.doc]

# QUESTIONNAIRE FOR GENETIC STUDY of HEAD-AND-NECK CANCER

**Name ___________________________________________________ Age ______ Sex Male Female**

**Date of Birth __________________________________ Primary Occupation_____________________**

**(month) (day) (year)**

**Address__________________________________________________________________________________**

**(street) (city) (state) (zip code)**

**Phone 1______________________________________Phone 2_____________________________________**

**(specify home or work)**

**Who would be likely to know your new address if you should move?**

**Name________________________Phone____________________Relationship________________________**

**Address__________________________________________________________________________________**

**(street) (city) (state) (zipcode)**

**Information about yourself:**

**If retired, provide longest job held____________________________________________________________**

**Ethnic background: African Caucasian Asian Pacific-Islander Hispanic**

**Native-American-Indian India-Indian Other _________________________________**

**[if mother (M) is different ethnic group from father (F), please note with an “M” and an “F”]**

**Do you smoke CIGARETTES/CIGARS/PIPE? ________ If yes, when did you begin? ________ (year)**

**and when did you stop? ___________(year) or are you still smoking? ______________________**

**On average, how many packs of cigarettes per day did you smoke over the entire time that you have been a cigarette smoker?**

**Less than ½** **½ 1 1½ 2 2½ 3 more than 3**

**How many cigars per day?_______________ How much pipe smoking per day?________(hours)**

**Is your spouse, or anyone else living in your home, a cigarette smoker? Yes__________No__________**

**How many people in your home are smokers? _________________**

**How many packs per day does the heaviest smoker smoke? ½ 1 1½ 2 2½ 3**

**Do you drink alcohol? No____ Yes____ Beer Wine Hard Liquor (whiskey, rum, vodka, etc.) (circle all**

**that apply)**

**2 or less 4 6 8 10 12 14 16 18 20 22 24 26 28 30 30+ drinks per week**

**Do you use snuff/chew tobacco? At least once a month At least once a week Every day Never**

**Do you drink coffee? ­­_____** **6 cups 4 cups 2 cups a day 1 or less cups a day None**

**Have you had 1 year or more occupational exposure (on the job)? If yes, please describe total number of**

**years:**

**Tar asphalt / blacktopping? ______________________________________________years**

**Tar roofing? _________________________________________________________years**

**Creosote-soaked wood? _________________________________________________years**

**Indoor exposure to motor engine exhaust? _______________________________years**

**Asbestos in the home or workplace? _______________________________years**

**Do you take supplemental vitamin C or vitamin E daily? If so, how many tablets of each, per day?**

**Vit C______________ Vit E_____________________________**

**How many days per week, or per month, do you eat charcoal-grilled meat (year round)? ___________**

**About once a month About once a week More than once per week**

**Information about your immediate family:**

**Do you have any family members (blood relatives) who’ve had any type of cancer? Yes No Don’t know**

**Do you have any family members who’ve had cancer of the mouth or throat? Yes No Don’t know**

**Or lung cancer? Yes No Don’t know**

**If YES to any family members with cancer, please complete the table below**

(Please note: half-brother or half-sister is a blood relative, but a step-brother or step-sister is not. There is no need to include cousins or second-cousins)

| **Mother** |  | **YES NO** |
| --- | --- | --- |
| **Father** |  | **YES NO** |
|  | **How many relatives do you have?** | **How many have had cancer?** |
| **Grandmothers** | **2** |  |
| **Grandfathers** | **2** |  |
| **Sisters or half-sisters** |  |  |
| **Brothers or half-brothers** |  |  |
| **Aunts (sisters of your mother or father)** |  |  |
| **Uncles (brothers of your mother or father)** |  |  |
| **Sons** |  |  |
| **Daughters** |  |  |
|  |  |  |

**FURTHER DETAILS FOR THE PARAMEDICAL PERSONNEL TO HELP ANSWER PATIENT’S QUESTIONS**

---**We want patients with HNSCC and a history of 1–40 pack-years (**highly sensitive, **HS). And patients with no cancer and a history of >80 pack-years (**highly resistant, **HR)**.

---**Race** If the patient is of mixed ethnic background, he/she can put “M” for one (or more) race(s) represented by the mother’s side of the family, and “F” for one (or more) race(s) represented by the father’s side of the family.

---**Smoking** **One** cigarette-pack-year = 20 cigarettes X 365 days = **7,300 cigarettes**. If the patient has smoked **at least 7,000 cigarettes (350 packs)** **over his/her lifetime**, we will consider this person a **smoker**. **Two cigars per week for 1 year = 100 cigars over his/her lifetime will be considered a smoker**. **Pipe-smoking once a day for 1 year** will be **enough to be considered a smoker**. Anything below these numbers we will consider a **nonsmoker** and exclude him/her from this study.

---**NOTE: Patients who have greater than 40, but less than 80, cigarette-pack-years of smoking history will be EXCLUDED from this study. If the history is “uncertain whether it’s 35 or 45 pack-years,” we should EXCLUDE such a patient from our study.**

---**Second-hand smoke** For second-hand smoke exposure as a risk factor, this will be studied as a co-variate in our final analysis of the data.

---**Alcohol** For drinking alcohol as a risk factor, this will be studied as a co-variate in our final analysis of the data.

---**Using snuff/chewing tobacco/snuff** For snuff-usage or tobacco-chewing as a risk factor, this will be studied as a co-variate in our final analysis of the data.

---**Coffee intake** This will be studied as a co-variate in our final analysis of the data.

---**Occupational chemicals** If a person has creosote-soaked railroad ties in their yard, this is not considered a significant exposure. If the person is in **the business** of working for years in and around creosote-soaked railroad ties, or works in a job where wood is soaked in creosote––such as climbing telephone poles––this is considered a significant exposure. This information his will be studied as a co-variate in our final analysis of the data.

---**Antioxidants** This will be studied as a co-variate in our final analysis of the data.

---**Grilled meat** For eating grilled meat as a risk factor, this will be studied as a co-variate in our final analysis of the data.

---**Family cancer history** Beyond the family members listed, we are not interested in the cancer history in cousins or second-cousins, etc. For family cancer history as a risk factor, this will be studied as a co-variate in our final analysis of the data.

**---Ultimately, we will examine these eight variance components as other risk factors (co-variates) by Multiple-Regression Analysis. Our staff will “rank” each of these co-variates as Zero (little or no contact), One (intermediate contact or exposure) or Two (high exposure) as a quick-and-simple means of eyeballing eight columns listed for all HS patients and HR subjects that we have ultimately collected.**
