# Supplementary material for: Head-and-neck squamous cell carcinoma risk in smokers: no association detected between phenotype and AHR, CYP1A1, CYP1A2, or CYP1B1 genotype
Source: Hum Genomics. 2016 Nov 28;10:39. doi: 10.1186/s40246-016-0094-y (PMC5127090; doi:10.1186/s40246-016-0094-y)
Supplement: Additional file 2: — Supplemental data online. (DOC 64 kb) [file 40246_2016_94_MOESM2_ESM.doc]

**SUPPLEMENTAL DATA ONLINE for:**

**Head-and-neck squamous cell carcinoma risk in smokers: No association detected between phenotype and *AHR*, *CYP1A1*, *CYP1A2*, or *CYP1B1* genotype**

**Lucia F. Jorge-Nebert, Ge Zhang, Keith M. Wilson, Zhengwen Jiang, Randall Butler, Jack L. Gluckman**,

**Susan M. Pinney,** and **Daniel W. Nebert**

**Supplemental Table S1**

Allele frequencies of *CYP1B1* SNPs and frequencies of inferred *CYP1B1* haplotypes in Europeans (EUR; N=1006 chromosomes) and Africans (AFR; N=1322 chromosomes)––based on the 1000Genomes Project, Phase 3 data

| **A** | | | | **EUR** | **AFR^1^** |
| --- | --- | --- | --- | --- | --- |
| **SNP ID** | **Reference allele** | **Alternative allele** | **Ancestral allele** | **q(alt)** | **q(alt)** |
| rs1056827 | C | A | A | 0.289 | 0.523 |
| rs10012 | G | C | C | 0.290 | 0.574 |
| rs162558 | T | C | T | 0.172 | 0.185 |
| rs2855655 | T | C | C | 0.634 | 0.266 |
| rs162557 | A | G | G | 0.762 | 0.778 |
| rs162556 | G | A | A | 0.500 | 0.965 |
| rs162555 | T | C | T | 0.175 | 0.166 |
| rs10175368 | C | T | C | 0.285 | 0.061 |

**B**

| **EUR** | | |
| --- | --- | --- |
| **Ranked order** | **Haplotype frequency** | **Haplotype sequence^2^** |
| 1 | 0.302 | c-g-T-C-G-g-T-C |
| 2 | 0.283 | A-C-T-C-G-A-T-t |
| 3 | 0.182 | c-g-T-t-a-g-T-C |
| 4 | 0.158 | c-g-c-t-G-A-c-C |
| 5 | 0.040 | c-g-T-C-a-A-T-C |
| 6 | 0.010 | c-g-T-t-a-A-T-C |
| 7 | 0.007 | c-g-c-t-G-g-c-C |
| 8 | 0.005 | c-g-c-t-a-g-c-C |
| **9** | **0.003** | **A-C-T-t-G-A-T-C** |
| 10 | 0.002 | c-g-T-C-G-A-T-t |
| 11 | 0.002 | c-g-T-C-G-g-c-C |
| 12 | 0.002 | A-C-T-C-G-A-c-C |
| 13 | 0.001 | A-C-T-C-G-A-T-C |
| 14 | 0.001 | c-g-c-C-G-g-T-C |
| 15 | 0.001 | c-C-T-C-G-g-T-C |
| 16 | 0.001 | c-g-c-t-a-A-c-C |

**C**

| **AFR^1^** | | |
| --- | --- | --- |
| **Ranked order** | **Haplotype**  **frequency** | **Haplotype sequence^2^** |
| **1** | **0.356** | **A-C-T-t-G-A-T-C** |
| 2 | 0.166 | c-g-c-t-G-A-c-C |
| 3 | 0.126 | c-g-T-t-a-A-T-C |
| 4 | 0.098 | A-C-T-C-G-A-T-C |
| 5 | 0.073 | c-g-T-C-a-A-T-C |
| 6 | 0.061 | A-C-T-C-G-A-T-t |
| 7 | 0.051 | c-C-T-t-G-A-T-C |
| 8 | 0.018 | c-g-T-C-G-g-T-C |
| 9 | 0.017 | c-g-c-t-G-A-T-C |
| 10 | 0.016 | c-g-T-t-a-g-T-C |
| 11 | 0.008 | c-g-T-C-G-A-T-C |
| 12 | 0.007 | A-C-T-C-a-A-T-C |
| 13 | 0.002 | A-C-c-t-G-A-T-C |
| 14 | 0.002 | c-g-T-t-G-A-T-C |
| 15 | 0.001 | c-g-T-C-a-g-T-C |

q(alt), frequency of alternative allele

**^1^** The AFR “super-population” in the 1000Genomes Project (Phase 3) comprises seven distinct African populations. Haplotype ACTTGATC shows significant frequency differences not only between AFR and EUR, but, even among the seven AFR populuations––the LWK (Luhya in Webuye, Kenya) has the highest frequency at 0.449 and ACB (African-Caribbeans in Barbados) the lowest frequency at 0.292. This finding makes the case/control analysis of this haplotype highly susceptible to a population-stratification artifact. Consequently, sample sizes of the non-Caucasian groups in our present HNSCC study are far too small to make any reliable conclusions.

**^2^**Ancestral alleles are shown in *upper case*.
